# Splicing Factors in Plant Abiotic Stress Responses: Regulatory Mechanisms and Perspectives

**DOI:** 10.3390/plants15152398

**Published:** 2026-08-05

**Authors:** Jiahui Guo, Qing Gao, Mengyu Zhou, Hongli Wang, Yijia Ruan, Xiaoyu Wang, Yujing Liu, Xinlei Du, Yishan Fu, Teng Zhang, Jintong Wang, Junfeng Zhang, Lei Cao

**Affiliations:** 1 College of Horticulture, Northeast Agricultural University, Harbin 150036, China; 13614671535@163.com (J.G.); 13039763390@163.com (Q.G.); zhoumengyu2025@163.com (M.Z.); whl22228888@163.com (H.W.); ryj2504019012@163.com (Y.R.); wxy766696114@163.com (X.W.); yujingliu0000@163.com (Y.L.); duxinlei0101@163.com (X.D.); fys01305611@163.com (Y.F.); zhangteng0012@163.com (T.Z.); wjt1482992725@163.com (J.W.); 2 School of Geography and Tourism, Harbin University, Harbin 150030, China; jfzhang@hrbu.edu.cn

**Keywords:** splicing factors, abiotic stress, regulatory mechanisms, breeding applications

## Abstract

Splicing factors, as core determinants of splice-site selection and dynamic spliceosome assembly, play pivotal roles in stress responses. This review systematically categorizes splicing factors involved in plant abiotic stress responses according to their functions as major spliceosomal components, dividing them into small nuclear ribonucleoproteins (snRNPs) and associated components, spliceosome assembly and disassembly factors, splicing regulatory factors, and proteins related to non-canonical RNA splicing. On this basis, we summarize their regulatory mechanisms of these factors under salt, drought, abscisic acid (ABA) signaling, temperature, and oxidative stresses. Through analyses across multiple species—including *Arabidopsis thaliana*, rice, maize, soybean, and wheat—we reveal both the evolutionary conservation and species-specific divergence of splicing-factor-mediated regulation. Currently, a large amount of research is still mainly at the transcriptome analysis or single phenotype validation stages, lacking in-depth analysis of direct targets, splicing isomer functions, and molecular mechanisms. Furthermore, current research is heavily concentrated on Arabidopsis, with relatively insufficient functional validation and breeding applications in crops such as maize and wheat. Despite substantial progress, several bottlenecks remain for translational applications in breeding, such as functional redundancy among splicing factor family members, growth penalties associated with overexpression, and tissue-specific and developmental-stage-dependent effects. To address these challenges, we discuss promising strategies, including CRISPR/Cas9-mediated splice-site editing, the use of inducible or tissue-specific promoters, and targeted modulation of upstream kinases, although extensive field trials and rigorous evaluations remain necessary. Collectively, this review provides a theoretical framework for understanding the roles of splicing factors in RNA-level regulation of plant stress adaptation and highlights their potential for breeding improvement.

## 1. Introduction

### 1.1. Classification and Working Mechanisms of Splicing Factors as Major Spliceosomal Components

According to functional hierarchy, splicing factors serving as major spliceosomal components involved in plant abiotic stress responses can be divided into four groups: core spliceosomal components, spliceosome assembly and disassembly-related factors, splicing regulatory factors, and non-canonical RNA splicing-related proteins. These groups coordinately constitute a splicing regulatory network at different levels.

The first category is core spliceosomal components. The spliceosome not only accurately identifies splice sites but also rapidly responds to regulatory signals, thereby enabling flexible splice-site selection during alternative splicing [1,2].

The second category includes spliceosome assembly and disassembly-related factors, such as SKIP and NTR1, which play critical roles in plant growth and development, environmental adaptability, and abiotic stress responses by participating in spliceosome assembly, functional regulation, or the disassembly cycle [3,4]. In addition, recent studies have identified accessory proteins that can activate transcription factors (e.g., bZIP60) through unconventional splicing under heat stress or endoplasmic reticulum stress, initiating the unfolded protein response [5].

The third category comprises splicing regulatory factors, including the SR protein family and other RNA-binding proteins. These factors, such as GauSR45a and OsSCR106, regulate pre-mRNA splicing of target genes and modulate splice-site selection, thereby influencing plant growth and development [6], hormone signaling pathways [6], and abiotic stress responses (including nutritional stress) [7], thus maintaining cellular homeostasis under stress conditions [7].

The fourth category includes non-canonical RNA splicing-related proteins involved in organellar intron splicing or auxiliary RNA regulatory protein functions.

Classifying splicing factors according to the above scheme facilitates the understanding of the splicing regulatory network in plant stress responses from three distinct levels—‘core spliceosomal components, splicing regulatory layer, and spliceosome dynamic regulatory layer’—and also enables a systematic appreciation of the functional division and synergistic relationships among different types of splicing factors.

### 1.2. Effects of Abiotic Stress on Plants and Research Progress on Splicing Factors

Abiotic stresses—including drought, high salinity, extreme temperatures, and alkalinity—are major causes of global crop yield losses, inflicting multiple injuries on plants such as ion toxicity, osmotic stress, oxidative damage, and metabolic disorders [8,9,10]. Studies across diverse plant species have demonstrated that stresses such as low temperature, drought, and salinity induce large-scale alternative splicing (AS) events and significantly affect related physiological processes [8,11,12,13,14]. Cold stress, in particular, exerts pronounced negative effects on the growth and development of many higher plants, restricting their geographic distribution and agricultural productivity [8]; for example, low temperature affects starch/sucrose metabolism in paper mulberry [8]. Drought stress disrupts plant growth by impairing water absorption and utilization efficiency [11]; it leads to reactive oxygen species (ROS) accumulation and membrane lipid oxidation in barley [11]; rice produces functional proteins via AS to cope with drought [13]; and in citrus, drought causes wilting and ROS accumulation [15]. Saline–alkali stress involves high pH and ionic imbalance, severely constraining crop growth and yield [9,10]. In summary, abiotic stresses such as drought, salinity, and low and high temperatures severely restrict plant growth and crop productivity through oxidative damage, ionic toxicity, and metabolic perturbations, and alternative splicing plays a significant regulatory role in these stress responses.

Alternative splicing (AS) is a crucial mechanism for regulating gene expression, enabling a single gene to generate multiple gene products with distinct biological functions, thereby expanding proteome diversity and functional complexity [16,17]. It is considered one of the key mechanisms by which plants rapidly adapt to environmental changes [13]. Splicing factors, by modulating splice-site selection, participate in the AS regulation of stress-related genes, enhancing transcriptomic plasticity and proteomic diversity [18,19]. Plant responses to abiotic stress involve multilayered networks, in which splicing factors, as core executors of post-transcriptional regulation, play pivotal roles. For example, under heat stress, the pre-mRNA of HsfA2 in *Arabidopsis* undergoes temperature-dependent alternative splicing, generating functionally distinct splice isoforms at 37 °C and 45 °C to finely modulate the intensity of the heat shock response [2,13]. Rice studies have also shown that AS facilitates plant adaptation to adverse environments by rapidly remodeling gene expression patterns [13]. With this deepening understanding, various splicing factors involved in splicing regulation have increasingly become research hotspots in plant stress biology.

### 1.3. Purpose of This Review

This review aims to systematically summarize the functional characteristics and regulatory mechanisms of different types of splicing factors and their alternative splicing products under abiotic stress, providing a theoretical reference for deciphering the RNA-based regulatory mechanisms of plant stress adaptation and for genetic improvement of crop stress tolerance. Currently, there has been extensive research on alternative splicing and its roles in stress responses in plants, but systematic synthesis focusing on the functional features, regulatory mechanisms, and recent progress of different splicing factor types under abiotic stress remains relatively fragmented. Therefore, this review focuses on the major splicing factors that play a role in plant abiotic stress responses, with emphasis on the roles and molecular mechanisms of core spliceosomal components, splicing regulatory factors, spliceosome assembly/disassembly factors, and non-canonical RNA splicing-related proteins in salt, drought, temperature stresses, and ABA signaling pathways. We further analyze current research problems and development trends, with the goal of providing a theoretical foundation for in-depth exploration of RNA-level regulatory networks in plant stress adaptation and for the genetic improvement of stress-resilient crops.

## 2. Composition of the Plant Splicing System and Functional Overview of Associated Regulatory Factors

**Figure 1:**
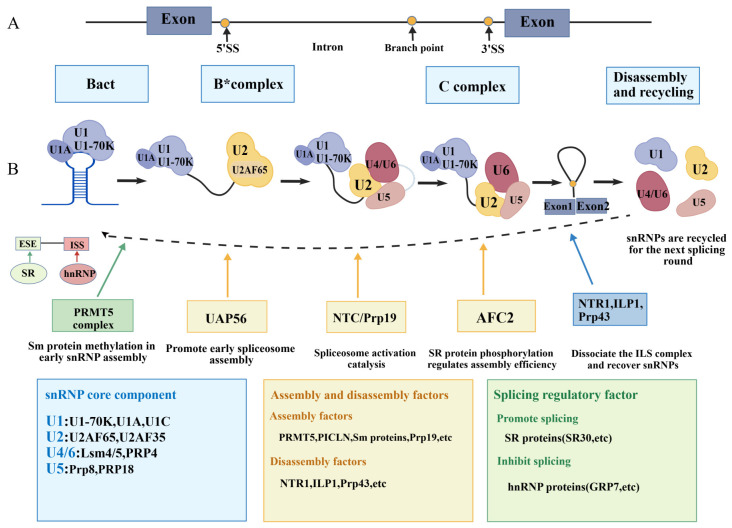
Dynamic assembly of the plant spliceosome and functional division of various splicing factors. (**A**) Schematic diagram of pre-mRNA structure, including the 5′ splice site (5′SS), branch point, and 3′ splice site (3′SS). (**B**) Dynamic assembly and functional roles of various factors. U1 snRNP first recognizes the 5′SS, followed by recruitment of U2 snRNP; subsequently, U4/U6 snRNP and U5 snRNP join to form the activated spliceosomal complex, which catalyzes intron excision and exon ligation. After splicing is completed, the intron lariat spliceosome (ILS) is disassembled, and snRNPs are recycled for the next round of splicing. Among these, PRMT5 and PICLN participate in early snRNP assembly by mediating Sm protein methylation; UAP56 promotes early spliceosome assembly; the NTC/Prp19 complex facilitates spliceosome activation; AFC2 modulates splicing efficiency through phosphorylation regulation of SR proteins; and Prp43 and NTR1 together with ILP1, are involved in spliceosome disassembly and snRNP recycling. SR proteins promote splice-site selection, whereas hnRNP proteins suppress splice-site selection. Image was created with the tools of BioGDP (https://BioGDP.com (accessed on 26 July 2026)).

Splicing is a dynamic and regulated process that relies on the continuous assembly, remodeling, and disassembly of the spliceosome—a large ribonucleoprotein complex composed of snRNAs and numerous proteins—which accomplishes pre-mRNA splicing through dynamic assembly. However, the regulation of alternative splicing in plants is not determined by a single splicing factor alone; rather, it constitutes a dynamic regulatory network involving core spliceosomal components, spliceosome assembly and disassembly factors, and splicing regulatory factors, as well as certain non-canonical RNA splicing-related proteins. Therefore, although splicing factors are important determinants of splice-site recognition, spliceosome assembly, and isoform formation, their functions depend on the coordinated actions at multiple regulatory levels (Figure 1A,B).

### 2.1. Core Spliceosomal Components

Small nuclear ribonucleoproteins (snRNPs) are the core catalytic components of precursor mRNA (pre-mRNA) alternative splicing (AS) in plants, primarily consisting of five snRNP particles—U1, U2, U4, U5, and U6—along with various auxiliary splicing factors [20]. Through the recognition of splice sites, coordination of spliceosome assembly, and catalysis of splicing reactions, snRNPs regulate plant development and responses to stress conditions such as drought and saline–alkali environments [2].

#### 2.1.1. U1 snRNP and Its Associated Factors

U1 snRNP is mainly responsible for recognizing the 5′ splice site (5′SS) of pre-mRNA, and this function has been validated across a wide range of organisms Refs [21,22] and participates in the ABA signaling pathway and multiple abiotic stress responses. Its core components include U1 snRNA, Sm core proteins, and specific proteins such as U1-70K, U1A, and U1C [23]. In plants, U1 snRNP is involved not only in constitutive splicing but also in stress-related alternative splicing. For example, studies have shown that by pairing with the 5′ splice site (5′SS) of *SPCH* pre-mRNA, U1 snRNP modulates intron retention in *SPCH* transcripts, thereby participating in the ABA signaling pathway and enhancing drought tolerance [24].

U1-70K is a specific protein of U1 snRNP. Studies have shown that it interacts with the AtDGCR14L protein and U1C, respectively, participating in the recognition of the pre-mRNA 5′SS [25,26]. The U1A protein also participates in 5′SS recognition [27] and interacts with U1C and U1-70K to facilitate in U1 snRNP assembly [27] (Table 1).

#### 2.1.2. U2 snRNP and Its Associated Factors

U2 snRNP is responsible for branch-point recognition during spliceosome assembly. Its core components include U2 snRNA, Sm-binding proteins, SF3a, and SF3b, with SF3b serving as a key factor in branch-point recognition and assembly [28,29]. U2 snRNP combines U2 snRNA and the U2 auxiliary factor (U2AF) to recognize the 3′SS. In mammals, U2AF consists of U2AF65 and U2AF35 [30]. In *Arabidopsis*, U2AF65 comprises U2AF65a and U2AF65b, two homologous proteins which are involved in the regulation of flowering time [14]. U2AF65b negatively regulates seedling growth and ABA-dependent inhibition of seed germination [31], whereas the AtU2AF65a protein is involved in the H_2_S-mediated salt stress response and enhances plant salt tolerance by regulating alternative splicing of salt-responsive genes [32].

Studies have shown that U2AF35b participates in the regulation of immune genes through interaction with SR45a [1]. The U2 snRNP-specific protein U2B″ binds to U2 snRNA and is involved in U2 snRNP assembly [33]. The SPF30 protein, as a U2 snRNP-associated splicing factor, interacts with U2AF65a and U2AF35b to regulate FLC splicing and flowering time [34] (Table 1).

#### 2.1.3. U4/U6 and U5 snRNP-Related Factors

U4/U6 and U5 snRNPs are primarily involved in the formation of the spliceosome catalytic center and also play important roles in dark and heat stress responses. The Prp8 protein, a core component of U5 snRNP, plays a critical role in the formation of the spliceosomal catalytic center [35]. The PRP18 protein is a U5-associated splicing factor mainly involved in the catalytic reaction of pre-mRNA splicing, specifically the exon ligation process [36], and its expression is upregulated under drought conditions [37]. RDM16, an Arabidopsis U4/U6 snRNP-associated protein (a homolog of human hPrp3), interacts with SPF30 to regulate flowering [34,38]. Moreover, RDM16 and its splice isoforms regulate the splicing of heat-responsive genes to enhance thermotolerance [39]. The PRP4 protein is a component of U4/U6 and is involved in the indirect regulation of dark and heat stress responses [40,41]. The LSm protein family (Sm-like proteins) are U6 snRNP-associated proteins [42] and play important roles in plant stress responses. Among these, the LSm4 protein is regulated by PRMT5-mediated symmetric dimethylation [43], while the LSm5 protein (also known as SAD1) is involved in maintaining U6 snRNA stability [44] (Table 1).

### 2.2. Spliceosome Assembly and Disassembly Factors

The spliceosome is not a static complex; rather, it undergoes continuous compositional changes during RNA substrate binding. Spliceosome assembly factors promote snRNP formation and early complex establishment; activation-related factors drive the transition to the catalytic state, and disassembly factors are responsible for post-splicing complex disassembly and snRNP recycling. Therefore, these dynamic regulatory factors collectively ensure the continuous progression of the splicing reaction and influence the splicing regulatory capacity of plants under developmental and abiotic stress conditions.

#### 2.2.1. Spliceosome Assembly Factors

Sm proteins are core components of various snRNPs [45]. The PRMT5 complex (also termed the methylosome), functioning as a pre-modification assembly factor for Sm proteins, plays a key role in the early stages of snRNP assembly. This complex consists of the PRMT5, PICLN, WD45, MEP50 and other proteins. PICLN forms a complex with PRMT5 and interacts with Sm proteins, and is thought to be involved in snRNP assembly in Arabidopsis [46,47]. The SMN complex component GEMIN2 protein is involved in spliceosome assembly and genetically interacts with PICLN to regulate alternative splicing [47]. These assembly factors coordinate Sm protein modification and snRNP assembly to jointly maintain proper spliceosome assembly and alternative splicing regulation.

Prp19 is a core component of the NTC (Nineteen Complex) that catalyzes spliceosome activation [48,49]. MAC3A and MAC3B, homologs of Prp19, are localized to the nucleus and interact with the HDA15 protein to co-regulate ABA-responsive intron retention [50,51]. SKIP, a Prp19 complex-associated protein, when mutated, leads to altered splicing patterns of ABA-responsive genes and also affects circadian-clock-related processes [52]. Additionally, SKIP regulates SWR1-C to participate in floral transition in Arabidopsis [53].

The DEAD-box RNA helicase UAP56 is an early spliceosome assembly factor with dual functions in RNA remodeling and RNA metabolism regulation. It comprises UAP56a and UAP56b and participates in the regulation of photomorphogenesis [54,55] (Table 1). In addition to canonical spliceosome assembly factors, proteins involved in the maintenance of snRNP homeostasis also indirectly regulate spliceosome assembly. USB1 is a 3′-terminal processing exonuclease of U6 snRNA and also participates in spliceosome assembly [56] (Table 1).

#### 2.2.2. Spliceosome Disassembly Factors

ILP1 and NTR1 are conserved ILS (intron lariat spliceosome) disassembly factors that play critical roles in the spliceosome disassembly stage [57]. NTR1, as an NTC-associated protein, participates in spliceosome disassembly and is responsible for the recycling of snRNPs after splicing, thereby maintaining the normal operation of the spliceosome cycle [58]. Studies have shown that NTR1 mutants are sensitive to high temperature, and heat stress inhibits its expression [3]. In addition, Prp43 is a DEAH-box helicase component and serves as a disassembly driver for the ILS spliceosome. Arabidopsis ILP1 and NTR1, as its associated disassembly factors, not only affect ILS spliceosome disassembly but also participate in miRNA biogenesis [57,59,60]. Mutations in both NTR1 and ILP1 lead to altered U6 snRNA stability and impaired ILS complex disassembly, consequently affecting overall splicing efficiency [61]. Collectively, these findings indicate that spliceosome disassembly factors not only participate in the RNA splicing cycle but also play important roles in plant stress responses and growth and development (Table 1).

### 2.3. Splicing Regulatory Factors

The core spliceosomal components are primarily responsible for establishing the basic architecture of the splicing reaction, whereas splicing regulatory factors promote or suppress spliceosomal site selection by recognizing specific sequences on pre-mRNAs, thereby regulating alternative splicing events such as exon skipping, intron retention, and 3’splice site selection.

The SR protein family represents the most widespread class of splicing factors; these proteins possess conserved domains, typically containing an RNA recognition motif (RRM) and a serine/arginine (SR)-rich C-terminal domain [62,63,64]. SR proteins are involved in the regulation of splicing of stress-responsive genes, and changes in their function can affect plant stress tolerance performance [65]. The SR proteins studied so far recognize exonic splicing enhancer sequences via RRM domains while recruiting spliceosomal components through RS domains [66]. Plant SR proteins can be classified into six subfamilies, among which SC, SR, and RS are homologous to human counterparts, while RS, RS2Z, and SCL are plant-specific [67]. In Arabidopsis, SR30 alters its splicing patterns under darkness and heat stress [40]. SCR106, as an SR-related protein, participates in the regulation of 3′ splice-site selection and stress response regulation [6].

hnRNP proteins typically recognize polypyrimidine tracts or U/G-rich sequences and are involved in exon skipping or intron retention [68,69]. Arabidopsis PTB1 and PTB2 are hnRNP family proteins that regulate intron splicing and exon selection, and are involved in development [70]. Previous studies have shown that the GRP7 protein can alter splice site selection and is also involved in the regulation of the circadian negative feedback loop [71,72]. In addition, it directly binds to *FLM* transcripts and promotes the production of the late-flowering transcript isoform *FLM-β* under low-temperature conditions [73].

Additionally, the ternary splicing factor complex composed of SWAP1, SFPS, and RRC1 in Arabidopsis participates in photomorphogenesis [74]; the ACINUS protein of the ASAP complex indirectly represses ABA responses by regulating the alternative splicing of ABH1 and HAB1, which are negative regulators of ABA signaling [75]. AFC2, a member of the LAMMER family kinases, participates in the phosphorylation regulation of SR proteins [76] and functions as a regulatory auxiliary factor.

In summary, alternative splicing is not a process independently determined by a single splicing factor, but, rather, a complex regulatory network jointly orchestrated by core spliceosomal components, spliceosome assembly and disassembly factors, and splicing regulatory factors. The core spliceosome executes the basic steps of the splicing reaction, whereas regulatory factors such as SR proteins and hnRNPs influence the final isoform composition by modulating splice site selection. Therefore, plant responses to environmental stress depend not only on changes in the expression of splicing factors, but also on the dynamic coordination of the entire RNA processing machinery (Table 1).

### 2.4. Non-Canonical RNA Splicing-Related Proteins

In addition to the nuclear spliceosome and its regulatory factors, plants also harbor a group of non-canonical RNA-related proteins. Although these proteins are typically not part of the canonical nuclear spliceosomal architecture, they serve as important components of the RNA processing regulatory network and play auxiliary roles in plant development and stress adaptation.

#### 2.4.1. Organellar RNA Splicing Factors

Distinct from other splicing factors, these proteins are localized to mitochondria or chloroplasts and participate in organellar intron splicing or form stress granules in response to environmental challenges. Their target RNA features include organellar intron boundary sequences or GAA-rich repeat sequences. In terms of organellar splicing, PPR-SMR1 and PPR-SPR2 proteins in maize interact to form a complex that mediates the splicing of multiple mitochondrial introns [77]; DEK2, DEK35, and EMP8 proteins are involved in the splicing of mitochondrial introns such as nad1, nad2, and nad4, thereby regulating seed development [78,79]. In *Arabidopsis*, the CFM9 protein and the mCSF1 protein participate in mitochondrial RNA splicing [76,80]. The rice OsNBL3 protein is involved in the splicing of mitochondrial nad5 intron 4 and affects complex I activity [81]. AtRUG3, an RCC1 family protein, is involved in the splicing of mitochondrial nad2 and maintains the assembly of Complex I [82]. AtPMH2 is a DEAD-box RNA helicase that maintains RNA structure and participates in mitochondrial splicing [83]. PORR (plant organellar RNA recognition) is a plant-specific RNA recognition domain; the AtWTF9 protein participates in the splicing of mitochondrial rpl2 and ccmFc [84], and the rice PORR-domain protein FSE5 is involved in mitochondrial nad4 splicing [85].

#### 2.4.2. Auxiliary RNA Regulatory Proteins

In stress responses, the STA1 protein (U5 snRNP-associated) is upregulated under cold stress, and its mutants exhibit cold sensitivity [86]. In Arabidopsis, RCF1 functions as a cold-inducible RNA helicase that regulates pre-mRNA splicing of cold-responsive genes and participates in low-temperature adaptation and cold tolerance regulation [87]. HIN1 is a plant-specific RNA-binding protein that binds to GAA repeat sequences and enhances plant osmotic stress tolerance by regulating the alternative splicing of pre-mRNA [88]. In addition, AtUBP1 is involved in stress granule formation, whereas the UBP1b mutant exhibits sensitivity to salt and osmotic stress [89,90]; the RH31 protein interacts with the TSN1 protein to form stress granules that regulate salt-responsive genes [91]. It has been demonstrated that some GRP family proteins, for example, GRP2, act as RNA chaperones and regulate mitochondrial gene expression under cold stress conditions [92], while APUM5, members of the PUM (Pumilio) protein family bind to the 3′UTR to regulate the expression of stress-responsive genes [93] (Table 1).

**Table 1:** Composition and associated regulatory factors of the plant splicing system.

Classification	Name	Species	Function	Reference
snRNP	U1 snRNA	Arabidopsis	U1 snRNA participates in the ABA pathway and by pairing with the 5′ splice site (5′SS) of *SPCH* pre-mRNA, U1 snRNP modulates intron retention in *SPCH* transcripts, thereby participating in the ABA signaling pathway and enhancing drought tolerance.	[24]
	U1-70K protein	Arabidopsis, cotton	The core protein of U1 snRNP interacts with AtDGCR14L protein and U1C, respectively, participating in the recognition of the pre-mRNA 5′SS.	[25,26]
	U1A, U1C proteins	Arabidopsis	The U1A protein participates in the recognition of the 5 ‘splice site (5’ SS) and interacts with U1C and U1-70K, jointly contributing to the assembly of the U1 snRNP.	[27]
	U2 snRNA	Arabidopsis	Core RNA component involved in 3’ SS site recognition.	[30]
	SPF30 protein	Arabidopsis	U2 snRNP-associated splicing factors interact with proteins such as U2AF65a and U2AF35b to regulate FLC gene splicing and flowering time.	[34]
	U2AF65a protein	Arabidopsis	AtU2AF65a is involved in the H_2_S-mediated salt stress response, enhancing plant salt tolerance by regulating alternative splicing of salt-responsive genes.	[32]
	U2AF65b protein	Arabidopsis	It negatively regulates seedling growth and inhibits seed germination following the application of ABA.	[31]
	U2AF35b protein	Arabidopsis	Interacts with SR45a and participates in the regulation of immune genes.	[1]
	PRP8 protein	Arabidopsis	The core components of U5 snRNP participate in the formation of the spliceosome ‘s catalytic active center.	[35]
	PRP18 protein	Corn	U5-related splicing factor mainly involved in the catalytic reaction of pre-mRNA splicing, and its expression can be increased under drought conditions.	[36,37]
	RDM16 protein	Arabidopsis	U4/U6 snRNP-associated proteins interact with SPF30.	[34,38]
	PRP4 protein	Arabidopsis	The components of U4/U6 participate in the indirect regulation of dark and heat stress.	[40,41]
	LSm4 protein	Arabidopsis	Regulation mediated by PRMT5-mediated symmetric dimethylation modification.	[43]
	LSm5 protein	Arabidopsis	Participates in maintaining the stability of U6 snRNA.	[44]
Splicing Assembly Factor	Sm Protein	Arabidopsis	The core components that constitute U1, U2, U4, and U5 snRNPs.	[45]
	PICLN protein	Arabidopsis	Interacts with PRMT5 and Sm proteins, participating in the assembly of Arabidopsis snRNP.	[47]
	GEMIN2 protein	Arabidopsis	Interacts genetically with PICLN to regulate alternative splicing.	[47]
	Prp19 protein	Arabidopsis	Core component of NTC, catalyzes spliceosome activation.	[48,49]
	SKIP protein	Arabidopsis	SKIP, a Prp19 complex-associated protein, when mutated, leads to altered splicing patterns of ABA-responsive genes and also affects circadian clock-related processes.	[52]
	UAP56a/UAP56b protein	Arabidopsis	Participate in the regulation of photomorphogenesis.	[54,55]
	USB1 protein	Arabidopsis	An exonuclease involved in the processing of the 3’ end of U6 snRNA, participating in spliceosome assembly.	[56]
Spliceosome dissociation factor	NTR1 protein	Arabidopsis	Participates in spliceosome dissociation and is responsible for the recycling of snRNPs after splicing.	[58]
	Prp43 protein	Arabidopsis	Components of the DEAH-box helicase participate in spliceosome dissociation; ILP1 forms a complex with NTR1 and Prp43, also contributing to spliceosome dissociation and miRNA biosynthesis.	[57,59,60]
	ILP1 protein	Arabidopsis	It is a conserved ILS dissociation factor and plays a key role during the spliceosome dissociation stage.	[57]
	AFC2 protein	Arabidopsis	The LAMMER family kinases are involved in the phosphorylation regulation of SR proteins.	[76]
Splicing regulatory factors	SR Protein Family	Various plants	The 6th subfamily (SC/SR/RS/RSZ/RS2Z/SCL) contains RRM+RS domains, promotes splicing, and recognizes exon splicing enhancers.	[62,63,64,66,67]
	SR30 protein	Arabidopsis	Changes splice patterns under dark and hot stress.	[40]
	SCR106 protein	Arabidopsis	SR-related proteins participate in regulating 3’ splicing site selection and are involved in stress response regulation.	[6]
	PTB1, PTB2 protein	Arabidopsis	An hnRNP family factor, regulating intron splicing and exon selection and participating in development.	[70]
	GRP7 protein	Arabidopsis	The hnRNP protein can alter the choice of splicing sites and also participate in regulating the biological clock negative feedback loop, and directly binds to *FLM* transcripts and promotes the production of the late-flowering transcript isoform *FLM-β* under low-temperature conditions.	[71,72,73]
	SWAP1, SFPS, RRC1	Arabidopsis	The ternary complex is involved in photomorphogenesis.	[74]
	ACINUS Protein	Arabidopsis	By regulating the variable splicing of ABH1 and HAB1, the negative regulatory factors for ABA signals are indirectly suppressed.	[75]
Organellar RNA splicing factor Auxiliary RNA regulatory protein	PPR-SMR1, PPR-SPR2 protein	Corn	PPR-SMR1 and PPR-SPR2 interact to form a complex that mediates the splicing of multiple mitochondrial introns.	[77]
	DEK2, DEK35, EMP8 protein	Corn	Participates in the splicing of mitochondrial introns such as nad1, nad2, and nad4, and regulates seed development.	[78,79]
	CFM9, mCSF1 protein	Arabidopsis	Participates in chloroplast and mitochondrial RNA splicing as well as rRNA processing.	[76,80]
	OsNBL3 protein	Rice	Participates in the splicing of mitochondrial nad5 intron 4 and influences the activity of complex I.	[81]
	RUG3 protein	Arabidopsis	Participates in the splicing of mitochondrial nad2 and maintain the assembly of Complex I.	[82]
	PMH2 protein	Arabidopsis	Maintains RNA structure and participates in mitochondrial splicing.	[83]
	UBP1 protein	Arabidopsis	Involves in stress particle formation, and UBP1b mutants show sensitivity to salt and osmotic stresses.	[89,90]
	TSN1, RH31 protein	Arabidopsis	Mutual interaction forms stress granules and regulates salt-response genes.	[91]
	HIN1 protein	Arabidopsis	By incorporating GAA repeat sequences and regulating alternative splicing of pre-mRNA, plant tolerance to osmotic stress is enhanced.	[88]
	APUM5 protein	_	Combining 3’UTR regulation with stress response gene expression.	[93]

Note: Selection criteria for factors listed in Table 1. The factors included in this table were supported by experimental evidence demonstrating their direct involvement in RNA splicing-related processes, including splice-site recognition, spliceosome assembly or disassembly, organelle intron splicing, or regulation of specific alternative splicing events. Candidates identified solely through transcriptome profiling, differential expression analysis, or genome-wide association studies without functional validation of their splicing-related roles were not considered experimentally validated splicing regulators. Such candidates are discussed as transcriptome-associated candidates rather than functional splicing factors. In addition, organelle RNA splicing factors and non-canonical RNA regulatory proteins are included because they contribute to RNA processing networks associated with splicing regulation. However, these proteins differ mechanistically from nuclear spliceosomal factors by acting primarily in organelle RNA maturation, RNA stability regulation, or stress granule-associated RNA control rather than participating directly in the canonical nuclear spliceosome.

## 3. Mechanisms of Plant Splicing Factors in Response to Different Abiotic Stresses

Informed by the concept of ‘systematic classification and evaluation of objects from multiple dimensions’ [94], as proposed in the Multidimensional Classification approach, we performed a systematic assessment of the existing factors using a framework based on the following seven dimensions: direct RNA-binding evidence, experimentally validated splicing events, functional differences between isoforms, genetic causality, overexpression phenotypes, transcriptomic associations, and field-level evidence. According to this framework, the factors are categorized as follows:

Category I (relatively complete causal chain) covers experimentally validated splicing events, functional differences between isoforms, and genetic causality, with at least one of either direct RNA-binding evidence or overexpression phenotypes, and supported by transcriptomic associations—that is, a full chain from splicing regulation to isoform function to genetic phenotype has been established.

Category II (partial causal chain) possesses experimentally validated splicing events and genetic causality but lacks functional differences between isoforms; or possesses functional differences between isoforms but lacks genetic causality.

Category III (phenotypic association) possesses genetic causality but lacks experimentally validated splicing events and functional differences between isoforms; or possesses transcriptomic associations but lacks genetic validation.

Category IV (transcriptomic association): only observational evidence at the transcriptomic level, without experimental validation.

In addition, genes such as *HvLHCA4.2*, *CiGI*, *RVE2*, *P5CS1*, *TaHSFA6e*, and *ZmHsf23* are not themselves splicing factors, but rather ‘target genes’ or ‘transcription factors’ that generate functionally divergent variants through stress-induced alternative splicing. These cases are treated as a separate category in this review to clearly distinguish them from bona fide splicing factors and avoid ambiguity in scope. This framework is applied uniformly throughout this section and the subsequent sections.

### 3.1. Salt Stress

Increased soil salinity poses a major threat to global agriculture and food security [95]. Under salt stress, splicing factors participate in stress adaptation by regulating the alternative splicing of downstream genes or through their own alternative splicing. Based on the seven dimensions described above, the evidence completeness of these factors falls into the following four categories: Category I (SR45a, AtDGCR14L, SRRM1L), Category II (AtU1A, RS33), Category III (OsSCR106, GsSCL30a, SC35), and Category IV (PtSCL30). In addition, some non-splicing factor genes (such as members of the bZIP transcription factor family) also participate in salt stress responses through stress-induced transcriptional activation, and they constitute different layers of the regulatory network together with splicing factors. All factors lack field validation. In the following, we evaluate each factor in the order of Categories I–IV and non-splicing factors.

Under salt stress, the alternative splicing pattern of the *SR45a* gene in *Arabidopsis* undergoes changes, producing two splice isoforms: the full-length SR45a-1a and the truncated SR45a-1b. The transcript levels of both variants are significantly upregulated following salt treatment. SR45a-1a directly interacts with the nuclear cap-binding protein CBP20, whereas SR45a-1b, although unable to interact with U1-70K, promotes the association between SR45a-1a and CBP20. At the target level, SR45a directly binds to the first intron of the *IDD14* pre-mRNA, modulating the isoform ratio of IDD14α to IDD14β. IDD14β forms non-functional heterodimers with *IDD14α*, competitively inhibiting the transcriptional activation activity of IDD14α on its downstream target QQS. Functional validation demonstrated that *SR45a* knockout mutants exhibit significantly enhanced salt tolerance, whereas overexpression of either *SR45a-1a* or *SR45a-1b* confers a salt-hypersensitive phenotype. The *sr45a cbp20* double mutant displays a phenotype similar to that of *cbp20*, indicating that both factors function in the same pathway to regulate salt stress responses [96].

AtDGCR14L is another splicing factor involved in salt stress responses in Arabidopsis. Its expression is continuously upregulated within 24 h under salt stress, exhibiting an expression pattern similar to that of salt-stress-responsive hypersensitive genes (*SOS1*, *SOS2*, and *SOS3*). AtDGCR14L interacts with the U1 snRNP component U1-70K in nuclear speckles via its highly conserved TWG motif (as confirmed by yeast two-hybrid and BiFC assays), participating in 5′SS recognition. Mutation of the TWG motif causes AtDGCR14L to become diffusely distributed throughout the nucleus, abolishing its interaction with U1-70K and its ability to complement the salt-sensitive phenotype of the mutant. At the target level, the pre-mRNAs of ABA signaling genes such as PIR1 and RAF10, as well as the core subunit *SWI3A* of the chromatin remodeling complex, exhibit increased intron retention in *AtDGCR14L* mutants, potentially leading to the production of truncated and inactive protein isoforms. In the case of *SWI3A*, intron retention results in the loss of the C-terminal leucine zipper domain, preventing assembly into a functional SWI/SNF complex. Expression of full-length SWI3A enhances salt tolerance, whereas the truncated isoform SWI3AΔC does not. Functional validation revealed that *AtDGCR14L* mutants exhibit significantly lower survival rates than wild-type plants on medium containing 200 mM NaCl, with more pronounced declines in chlorophyll fluorescence; complementation with *AtDGCR14L* restores salt tolerance, whereas the TWG mutant fails to do so [25].

**Figure 2:**
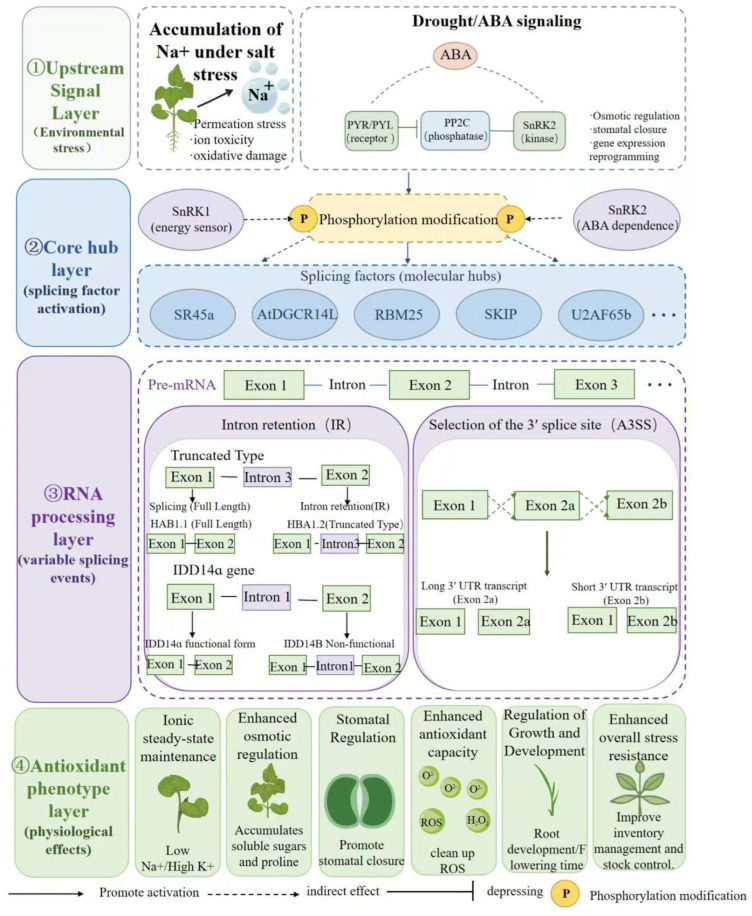
Splicing factor-mediated core signaling regulatory network in response to salt/drought/ABA stresses. The network is organized into four hierarchical layers: ① upstream environmental stress signaling layer; ② core splicing factor hub layer; ③ pre-mRNA alternative splicing processing layer; and ④ physiological stress-tolerance phenotype output layer. Different splicing factors regulate stress-adaptive physiological processes—including ion homeostasis, stomatal closure, and ROS scavenging—by mediating alternative splicing events such as intron retention and 3′ splice site selection. Image was created with the tools of BioGDP (https://BioGDP.com (accessed on 26 July 2026)).

Salt stress activates SnRK1 kinase, which phosphorylates nine serine/threonine sites on the SRRM1L protein (Figure 2 ②). Phosphorylation promotes the aggregation of SRRM1L in nuclear speckles, where it interacts with the U1 snRNP component U1-70K through its RS domain to participate in early spliceosome assembly; mutation of the phosphorylation sites leads to diffuse distribution and loss of interaction with U1-70K. SRRM1L directly binds to exon 1 of the NFYA10 pre-mRNA via its PWI domain (as confirmed by RIP assay), regulating its alternative splicing to produce two variants: the functional NFYA10.1 and the non-functional NFYA10.3. NFYA10.1 encodes a functional *NFYA10* transcription factor, whereas NFYA10.3 is a truncated protein degraded via the nonsense-mediated decay (NMD) pathway. Functional validation demonstrated that srrm1l mutants (including both T-DNA insertion and CRISPR/Cas9 knockout alleles) are salt-sensitive, and overexpression of *SRRM1L* enhances salt tolerance, whereas the phospho-deficient mutant SRRM1L(9A) does not [97].

AtU1A is involved in the splicing regulation of *ACO1* pre-mRNA (as confirmed by RNA-IP assay), AtU1A affects reactive oxygen species levels by regulating the alternative splicing of *ACO1*, and *AtU1A* mutants are hypersensitive to salt stress [27]. Loss of function of rice *RS33* leads to enhanced salt stress sensitivity: the rs33 mutant showed significantly inhibited seed germination and seedling growth under 100 mM NaCl treatment; RNA-seq analysis revealed that approximately 600 genes underwent differential splicing in the rs33 mutant, predominantly involving intron retention (IR) events, whereas the functional differences of the isoforms of specific targets remain to be identified [98]. Rice *OsSCR106* regulates the splicing patterns of downstream genes (likely primarily through influencing 3′SS selection), and *OsSCR106* mutants are highly sensitive to salt stress, but their specific target genes have not yet been identified [6]. In addition, under alkaline stress, wild soybean *GsSnRK1* phosphorylates six serine residues of GsSCL30a, enhancing its splicing activity on its own third intron. GsSCL30a interacts with U1-70K to participate in 5′SS recognition, thereby promoting correct splicing of target genes and improving sodium bicarbonate stress tolerance [10]. Poplar SC35 mediates the splicing of *bZIP49*, and under salt stress, the bZIP49S isoform negatively regulates the expression of the AKT1 potassium channel [99]. Heterologous overexpression of the splicing factor *PtSCL30* reduces salt stress tolerance in Arabidopsis [100]. In cotton, 13 SR protein-encoding genes undergo differential splicing under salt stress [101].

In addition to the splicing factors mentioned above, some non-splicing factor genes also participate in salt stress responses through stress-induced transcriptional activation. The bZIP transcription factors are among the most representative of this category. Under salt stress, bZIP transcription factors bind to ABA-responsive elements (ABREs) in the promoters of target genes, activating the expression of downstream stress-responsive genes and thereby enhancing plant salt tolerance [102]. Unlike splicing factors, the evidence for non-splicing factors such as bZIP under salt stress is primarily reflected in direct DNA binding (as confirmed by yeast one-hybrid assay), genetic causality, overexpression phenotypes, and transcriptomic associations, but lacks evidence in dimensions such as validated splicing events and functional differences between isoforms; moreover, all these factors lack field validation.

Among the factors described above, Category I factors (*SR45a*, *AtDGCR14L*, *SRRM1L*) have established a complete causal chain from splicing to isoform function to genetic phenotype. Category II factors (AtU1A and RS33) have been validated in splicing events and exhibit genetic causality, but lack evidence for functional differences between isoforms. Category III factors (*OsSCR106*, *GsSCL30a*, *SC35*) primarily face challenges such as unknown target genes or missing genetic causality. The Category IV factor (*PtSCL30*) remains only at the level of transcriptomic associations or heterologous overexpression. In addition, the non-splicing factor bZIP transcription factors participate in salt stress responses by binding to ABRE elements and activating downstream gene expression; however, the upstream splicing regulatory mechanisms remain poorly understood, and field validation is absent in all cases.

### 3.2. Drought Stress

Drought is one of the most significant factors affecting plant growth, development, survival, and crop productivity, posing a major threat to sustainable agricultural development. Under drought stress, plants reprogram gene expression through large-scale alternative splicing to cope with the damage caused by water deficit. Research on splicing regulation under drought stress is relatively limited compared with that under salt stress, with most studies concentrating on the identification of alternative splicing (AS) events at the transcriptomic level rather than on functional characterization of specific splicing factors. All factors lack field validation. In the following, we evaluate each factor in the order of Category II, Category IV, and non-splicing factors.

In maize, ZmMBF1 likely participates in spliceosome-related processes by directly binding to U1 snRNA and interacting with the U1 snRNP component U1-70K. In *ZmMBF1* knockout mutants, the exon splicing efficiency of *ZmWRKY30* and *ZmMYBR63* is significantly altered, indicating that ZmMBF1 regulates the splicing of downstream drought-tolerance-related genes. Functional validation demonstrated that *ZmMBF1*-overexpressing plants exhibit significantly enhanced drought tolerance, whereas knockout mutants display a drought-sensitive phenotype with substantially lower survival rates than wild-type plants. Comprehensive experimental analyses further indicate that *ZmMBF1* positively regulates drought tolerance in maize by modulating the splicing of drought-responsive genes [103].

*PRP18* shows drought-induced expression at the transcriptomic level [37], but functional validation and target identification are lacking.

In addition to splicing factors, certain non-splicing factor genes also produce functionally differentiated protein variants through drought-induced alternative splicing, thereby participating in drought responses. For example, the barley light-harvesting complex protein gene *HvLHCA4.2* generates the *HvLHCA4.2b* variant through drought-induced A3SS (alternative 3′ splice site) selection (Figure 2 ③). This variant exhibits enhanced interaction interfaces with LHCA1. Double silencing of both *HvLHCA4.2a* and *HvLHCA4.2b* renders plants drought-sensitive, manifesting as reduced water retention capacity, ROS accumulation, and elevated malondialdehyde (MDA) content, indicating that HvLHCA4.2b plays an important role in maintaining photosynthetic system stability and ROS homeostasis under drought conditions [11]. In lemon, the clock-regulatory protein gene *CiGI* undergoes intron retention, producing a truncated variant *CiGIβ*, whereas *CiGIα* is the normally spliced full-length variant. Overexpression of *CiGIα* enhances drought sensitivity, whereas CiGIβ exhibits no significant phenotypic effect, suggesting functional divergence between the different variants generated by *CiGI* alternative splicing in drought responses [15].

Overall, ZmMBF1 (Category II) has experimental validation but lacks functional differences between isoforms, transcriptomic associations, and field validation. *PRP18* (Category IV) shows drought-induced expression at the transcriptomic level [37], but lacks functional validation and target identification. *HvLHCA4.2* and *CiGIα* are not splicing factors themselves; rather, they produce functionally divergent protein variants through drought-induced alternative splicing of their own transcripts. Cross-species comparisons reveal that intron retention is also the predominant AS event type under drought stress, and drought-stress-induced AS reprogramming exhibits pronounced tissue specificity—in maize, the responses in leaves and ears are considerably stronger than those in tassels—suggesting that the functions of splicing factors in drought responses are tissue-dependent.

### 3.3. ABA Signaling Pathway

ABA activates downstream stress responses through the receptor-PP2C-SnRK2 signaling module. Multiple splicing factors (RBM25 [104], *SmEb* [105]) finely modulate stress response intensity by regulating the alternative splicing of key genes in the ABA signaling pathway (Figure 2 ①). The category distribution is as follows: Category I comprises (SmEb and RBM25), and Category II comprises (SKIP, SR45, and AtU2AF65b). Notably, both RBM25 and SmEb target *HAB1*, while both SKIP and AtU2AF65b are involved in *ABI5* regulation, suggesting functional convergence of splicing factors in the ABA pathway. All factors lack field validation. In the following, we evaluate each factor in the order of Category I, Category II, and non-splicing factors.

In *Arabidopsis*, ABA treatment induces upregulation of *RBM25* expression. RBM25 binds to the last intron of *HAB1* (as confirmed by RIP assay) to regulate its alternative splicing, generating two variants, HAB1.1 and HAB1.2 [104]. HAB1.1 can bind to and dephosphorylate SnRK2.6, thereby suppressing ABA signaling, whereas HAB1.2, which contains a premature termination codon (PTC) resulting in a truncated protein, is presumably unable to dephosphorylate SnRK2.6 [106]. In *rbm25* mutants, the HAB1.2/HAB1.1 ratio is elevated, leading to sustained activation of SnRK2.6 and consequent ABA hypersensitivity [104] (Figure 2 ④). Overexpression of *HAB1.1* complemented the ABA-hypersensitive phenotype of the *rbm25* mutant; overexpression of *RBM25* also restored the HAB1.2/HAB1.1 ratio and ABA sensitivity to wild-type levels [104].

SmEb similarly participates in ABA signaling by regulating the alternative splicing of *HAB1*. In *smeb* mutants, accumulation of the *HAB1.2* (truncated) transcript in seedlings increases, whereas that of *HAB1.1* (full-length) decreases. *SmEb* mutants exhibit an ABA-hypersensitive phenotype during seed germination and seedling growth [107]. Mutations in *abi3, abi4*, and *abi5* all attenuate the ABA hypersensitivity of the *smeb* mutant. The ABA-hypersensitive phenotype of the *smeb* mutant is not caused by altered ABA content, indicating that SmEb modulates ABA responses through regulation of ABA signal transduction rather than ABA biosynthesis [107].

SKIP regulates the splicing of ABA receptor genes *PYL7/PYL8*, transcription factors *ABF1/ABF2*, and multiple *PP2C* genes (as verified by RIP analysis); intron retention leads to the production of non-functional truncated proteins, affecting the PP2C-mediated negative feedback regulation of ABA signaling, thereby conferring an ABA-insensitive phenotype on the skip-1 mutant [52].

SR45 is an SR-like splicing factor involved in the regulation of ABA signaling and drought stress responses. Through RIP-seq, more than 4000 RNA targets bound by SR45 were identified, among which ABA signaling pathway genes include the PP2C phosphatase *HAB1*, the ABA receptor *PYR1*, and the protein kinase *SnRK2.2*. In the *sr45* mutant, the total transcript level of *HAB1* was significantly reduced, whereas the level of intron-retaining isoforms was increased. Functional assays demonstrated that the *sr45* mutant exhibited exacerbated primary root growth inhibition under ABA treatment, displaying an ABA-hypersensitive phenotype. Furthermore, water loss from detached leaves of the *sr45* mutant was significantly faster than that of the wild-type, indicating that SR45 plays a positive regulatory role in drought stress responses [108].

*AtU2AF65b* expression is also upregulated in response to ABA treatment. AtU2AF65b interacts with U2AF35a/b via its RRM domain, likely participating in 3′SS recognition. In *atu2af65b* mutants, intron retention of *ABI5* and *FLC* pre-mRNAs is increased, and splicing efficiency is reduced, resulting in significantly decreased transcript levels of *ABI5* and *FLC*. ABI5 acts as a positive transcriptional regulator of *FLC*; the decline in its expression caused by aberrant splicing subsequently attenuates *FLC* transcriptional activation. Functional validation demonstrated that *atu2af65b* mutants flower early under both long-day and short-day conditions and are insensitive to exogenous ABA treatment that promotes flowering [14].

In addition to splicing factors, non-splicing factor genes also participate in ABA signaling regulation through ABA-induced alternative splicing of their own transcripts. In rapeseed, ABA treatment induces an increase in the number of AS events at the genomic level [109]. In rapeseed (*Brassica napus*), the *BnABF4L* gene undergoes an alternative 3′ splice site (A3SS) event under stress conditions, generating three transcript variants, *V1*, *V2*, and *V3*. The 5′ untranslated region (UTR) of *V1* contains seven upstream open reading frames (uORFs) that strongly repress the translation of the primary ORF (pORF), whereas the 5′ UTRs of *V2* and *V3* lack such inhibitory uORFs, thus allowing efficient translation. Overexpression experiments demonstrated that *V2* overexpression enhances ABA sensitivity and heat tolerance but inhibits growth, whereas *V3* enhances stress tolerance with relatively minor effects on growth and development. Under stress conditions, the splicing pattern of *BnABF4L* shifts toward *V3*, thereby circumventing uORF-mediated translational repression [110].

In summary, the RBM25-*HAB1* module establishes a relatively complete causal chain from ABA signaling to isoform function and phenotypic outcome. Category I factors encompass direct RNA-binding evidence and transcriptomic associations, yet lack field validation. SmEb, also classified as Category I, functions as a core Sm protein of the spliceosome and participates in ABA signaling through regulating alternative splicing of *HAB1*, but it lacks direct RNA-binding evidence. Both SmEb and RBM25 target *HAB1*, but whether they function synergistically remains unclear. Both SmEb and RBM25 target *HAB1*, but whether they act synergistically remains unclear. SKIP (Category II) regulates the splicing of multiple ABA pathway genes and covers direct RNA-binding evidence, experimentally validated splicing events, genetic causality, and transcriptomic associations, but lacks functional validation between isoforms and overexpression phenotypes. SR45 (Category II) has identified 43 target genes in the ABA signaling pathway through RIP-seq. SR45 covers direct RNA-binding evidence, experimentally validated splicing events, genetic causality, and transcriptomic associations, but lacks functional validation between isoforms and overexpression phenotypes. AtU2AF65b (Category II) participates in ABA-mediated flowering regulation through the splicing of *ABI5* and *FLC*, covering experimentally validated splicing events, genetic causality, and transcriptomic associations, but lacks direct RNA-binding evidence, functional validation between isoforms, and overexpression phenotypes. In rapeseed (*Brassica napus*), *BnABF4L* generates three variants through A3SS, and under stress conditions, its splicing shifts toward *V3*, thereby enhancing stress adaptability; however, the upstream splicing factors involved remain unclear. All of the above factors lack field validation.

### 3.4. Regulatory Network of as Under Cold Stress

Studies have demonstrated that low-temperature stress induces large-scale and rapid AS reprogramming [111], with hundreds of ‘early AS genes’ regulated exclusively through AS, which is essential for cold tolerance [16,112]. Under cold stress, the category distribution is as follows: Category II comprises STA1, PICLN, RS33, and AtSF1; Category III comprises OsSCR106. All factors lack field validation. In the following, we evaluate each factor in the order of Categories II through IV, followed by non-splicing factors.

Cold stress induces upregulation of *STA1* expression, which encodes a *U5 snRNP*-associated protein involved in spliceosome assembly. Under cold stress, RNA hybridization analysis showed that the pre-mRNA of the cold-inducible gene *COR15A* undergoes intron retention in *sta1* mutants, producing unspliced transcripts. Functional validation revealed that *sta1* mutants exhibit a cold-sensitive phenotype, with all mutant plants dying at 4 °C while wild-type plants survived; furthermore, *sta1* mutants are hypersensitive to ABA, and *STA1* transcripts themselves show enhanced stability in the mutant background. The above evidence indicates that STA1 regulates the splicing of *COR15A* under cold stress, but the functional differences between the two splicing isoforms of *COR15A* have not yet been verified [86].

Additionally, low-temperature stress induces upregulation of *PICLN* expression. PICLN participates in snRNP assembly and splicing regulation through interactions with PRMT5 and Sm proteins. *Picln* mutants exhibit severely inhibited root growth at low temperatures: Transcriptome analysis showed that at the normal growth temperature of 22 °C, 611 splicing defects involving 554 genes (including all differential splicing types such as intron retention, exon skipping, and alternative 5′/3′ splice sites) were detected in *picln* mutants; upon exposure to 10 °C for 24 h, the total number of splicing defects increased significantly to 1215 events involving 1020 genes. This indicates that *PICLN* plays a substantially enhanced role in maintaining genome-wide splicing fidelity under low-temperature conditions [47]. RT-PCR verified splicing defects in a subset of genes. Functional validation indicated that the picln-1 mutant exhibited severely inhibited root growth under low temperature; double mutants of *picln* with *prmt5*, *gemin2*, or *sme1* are all embryonic lethal. PICLN interacts with multiple Sm proteins. PICLN also exhibits a sensitive phenotype under salt stress [47].

Meanwhile, the rice SR protein RS33 regulates the splicing of a large number of genes under cold stress. Transcriptome analysis revealed that the rs33 mutant, compared with the wild-type, exhibited numerous differential splicing events at both 3 h and 6 h after cold treatment, predominantly through intron retention (IR) events. RT-PCR verified that intron retention events in a subset of genes (e.g., Os03g02610, Os05g04580, etc.) were significantly enriched in the rs33 mutant after cold treatment. Functional validation showed that the rs33 loss-of-function mutant exhibited increased sensitivity to both cold and salt stress [98].

AtSF1 is responsible for recognizing the branch point sequence within introns and is a key factor in pre-mRNA splicing. EMSA confirmed that AtSF1 directly binds to the branch point sequence containing the 5′-CU(U/A)AU-3′ motif. The atsf1 mutant exhibits delayed chloroplast development under cold stress, manifested as impaired maturation of plastid ribosomal RNA; transcriptome analysis revealed that multiple genes in the atsf1 mutant show aberrant expression due to defective intron splicing [27].

Cold stress significantly affects *OsSCR106* expression in rice, and *scr106* mutants exhibit severely inhibited root and shoot growth at low temperatures, indicating that OsSCR106 also plays an important role in cold stress responses; complementation with OsSCR106 rescues the stress-sensitive phenotype. Genome-wide splicing analysis, mainly under salt stress conditions, suggests that OsSCR106 may regulate its targets primarily through modulating 3′ splice site selection; however, the specific target genes and the corresponding splicing events have not yet been experimentally validated [6]. In paper mulberry under cold stress, A3 events are the most abundant among the seven AS event types. Differentially spliced genes are significantly enriched in starch/sucrose metabolism and circadian rhythm pathways. Among 41 SR protein genes, 29 undergo AS and 11 are differentially expressed. Genes undergoing AS exhibit significantly higher methylation levels in the CpG context [8].

*RVE2* and *P5CS1* are not splicing factors but represent cases of ‘gene self-alternative splicing’—the former encodes an MYB transcription factor [113] and the latter encodes proline synthetase [114]—exemplifying scenarios in which genes themselves undergo alternative splicing under low-temperature conditions to generate functionally differentiated variants.

In summary, STA1 (Category II) establishes a relatively complete chain from cold-induced expression and splicing to target splicing events and cold-sensitive phenotypes, although the functional differences between the two *COR15A* splice isoforms have not been validated, and there is a lack of direct RNA-binding evidence and overexpression phenotypes. PICLN (Category II), as a spliceosome assembly factor, regulates snRNP assembly, but its directly regulated target transcripts remain unclear. RS33 (class II) RT-PCR has verified some IR events, and the mutant is sensitive to cold and salt stress, but the functional differences among the splicing isoforms of its target genes have not been elucidated; AtSF1 (class II) EMSA has confirmed its direct binding to the branch point sequence, yet the target splicing events under cold stress remain to be systematically identified. The specific targets and splice variant functions of OsSCR106 (Category III) under cold stress have also not been identified. All factors lack field validation. Cross-species comparison reveals that cold-induced AS studies in *Arabidopsis* are relatively systematic; the functions of rice OsSCR106 and RS33 have been validated but mechanistic depth remains insufficient; studies in paper mulberry suggest the involvement of DNA methylation in cold stress AS regulation, an epigenetic dimension less explored in *Arabidopsis*. Across species, upregulation of splicing factor expression and maintenance of splicing fidelity under low-temperature stress appear to be common features, while differences exist in response kinetics and predominant AS event types among species.

### 3.5. Heat Stress

**Figure 3:**
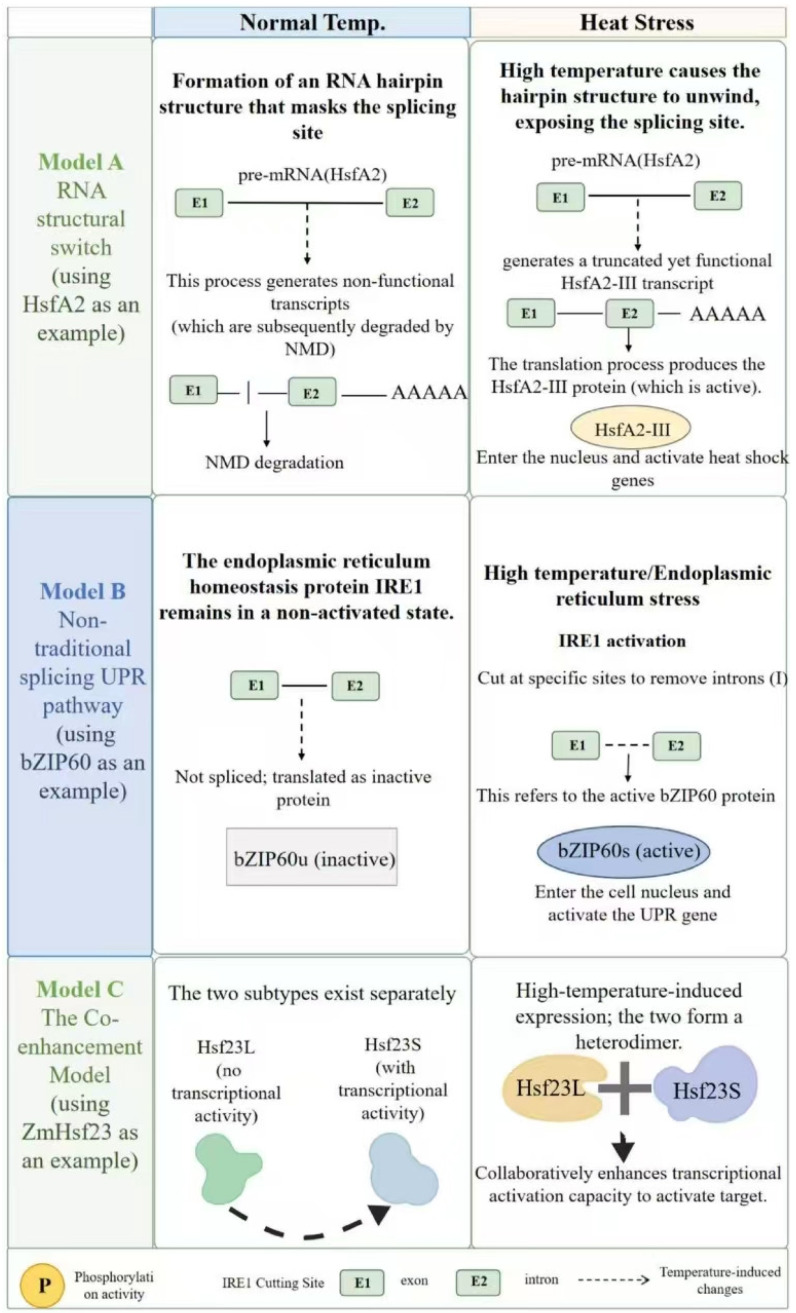
Temperature-dependent alternative splicing and heat stress response mechanisms. Model (**A**): The HsfA2 pre-mRNA relies on a temperature-responsive RNA stem-loop structural switch to regulate the generation of functional splice isoforms. Model (**B**): ER stress activates IRE1-mediated unconventional splicing of bZIP60 to initiate the unfolded protein response. Model (**C**): Two splice isoforms of maize *ZmHsf23* form heterodimers that synergistically enhance the transcriptional activation of heat shock genes. Image was created with the tools of BioGDP (https://BioGDP.com (accessed on 26 July 2026)).

Heat stress exerts profound effects on plant growth and development [115]. Under heat stress, plant splicing factors primarily function through two modes: upregulation of their own expression, phosphorylation modification, or subcellular relocalization to regulate the splicing of target genes; and temperature-dependent alternative splicing of heat shock transcription factor genes (e.g., *HsfA2*, *TaHSFA6e*, *ZmHsf23*) themselves, generating splice isoforms with distinct functions (Figure 3A). Under heat stress, the category distribution is as follows: Category I comprises the RS2Z35/RS2Z36 functional group and CYP18-2, and Category II comprises IRE1b. All factors lack field validation. In the following, we evaluate each factor/functional group in the order of Category I, Category II, and non-splicing factors.

In Arabidopsis, heat stress regulates CYP18-2, and the CYP18-2 protein translocates from the nucleoplasm to nuclear speckles and cytoplasmic granules, where it interacts with MAC3A and MAC3B and tightly associates with U5 and U6 snRNAs. The interaction was confirmed by RNA-IP assay. In *cyp18-2* mutants, intron retention levels of *HSFA2*, *HSFA7a*, and *HSP101* are significantly reduced. *HSP101-IR* and *HSFA2-III-IR* can be translated into truncated proteins; HSFA2-III interacts with HSFA1a and HSP90.1 and participates in heat stress responses. Functional validation demonstrated that *cyp18-2* mutants exhibit significantly lower survival rates than wild-type plants after heat stress at 47 °C [116]. Genome-wide transcriptome analysis revealed that the *cyp18-2* mutation affects alternative splicing of heat-stress-responsive genes, particularly intron retention (IR)—the levels of intron retention in heat-stress-related genes were significantly reduced in the *cyp18-2* mutant. Furthermore, complementation assays further confirmed the critical function of CYP18-2 in heat stress tolerance [116].

Tomato RS2Z35 and RS2Z36 are plant-specific SR proteins that recognize target RNA binding by engaging GGAG motifs, this direct interaction was confirmed by iCLIP assay, mediating approximately 50% of heat-sensitive AS events (with intron retention being the most abundant type). RS2Z35 is constitutively expressed, whereas RS2Z36 is heat-inducible. In *rs2z35rs2z36* double mutants, intron splicing of *HSFA2* is enhanced, resulting in reduced levels of functional HSFA2-I protein and significantly diminished both basal and acquired thermotolerance [117].

Heat stress activates ER-localized IRE1b, which catalyzes the unconventional splicing of *bZIP60* pre-mRNA. This splicing generates two variants: bZIP60(u) (containing a transmembrane domain and localized to the ER) and bZIP60(s) (lacking the transmembrane domain and translocating to the nucleus). Upon nuclear entry, bZIP60(s) participates in activating UPR target genes (e.g., *BIP3*), contributing to heat stress adaptation. In *ire1b* mutants, *bZIP60* splicing is significantly reduced and *BIP3* induction is impaired [5] (Figure 3B).

Furthermore, alternative splicing of non-splicing factor genes also participates in heat stress responses. Wheat *TaHSFA6e* generates two variants, TaHSFA6e-II and TaHSFA6e-III, through heat-induced AS; the latter exhibits stronger transcriptional activation activity due to the formation of an AHA domain at its C-terminus, and knockout mutants are heat-sensitive [118]. High temperature induces widespread AS in maize [119]. Maize *ZmHsf23* produces Hsf23L (containing a mini-exon) and Hsf23S through AS; Hsf23L interacts with Hsf23S to enhance their transcriptional activation of *sHSPs* and *TIL1*. Single knockout of *hsf23L* or double knockout both result in heat sensitivity; overexpression of Hsf23S enhances thermotolerance, while co-overexpression of both further improves thermotolerance [120] (Figure 3C).

In summary, under heat stress, RS2Z35/RS2Z36 and CYP18-2 establish a relatively complete causal chain (Category I), covering direct RNA-binding evidence, experimentally validated splicing events, functional validation between isoforms, genetic causality, and transcriptomic associations, but lack overexpression phenotypes and field validation. IRE1b-bZIP60 (Category II) represents a special mechanism of non-canonical splicing, whereby IRE1b-catalyzed splicing of *bZIP60* mRNA generates functionally distinct protein variants, covering experimentally validated splicing events, functional validation between isoforms, and genetic causality. CYP18-2 (Category I) is a splicing factor belonging to the cyclophilin family, and the evidence chain for CYP18-2 under heat stress is relatively complete, supported by experimental data on heat-induced expression, subcellular relocalization, interactions with spliceosomal components, regulation of target intron retention, and heat-sensitive mutant phenotypes. It covers experimentally validated splicing events, functional validation between isoforms, genetic causality, overexpression phenotypes, and transcriptomic associations, but lacking direct RNA-binding evidence and field validation. Temperature-dependent alternative splicing of heat shock transcription factors themselves has been observed across multiple species (*Arabidopsis* HsfA2, tomato HsfA2, wheat TaHSFA6e, maize ZmHsf23), suggesting that this mechanism may be evolutionarily conserved. *TaHsfA2-7* is induced by high temperature and actively responds in thermotolerance regulation [121]. Structural studies on HsfA2 RNA [122] have focused on the molecular mechanisms of AS, providing structural foundations for understanding temperature-dependent splicing regulation, although they do not directly address functional validation of splicing factors. All factors lack field validation. In heat stress, although mechanistic studies are relatively thorough, the translation from model plants to crop breeding still requires further strengthening.

### 3.6. Oxidative Stress and Hypoxia Stress

Reactive oxygen species (ROS) are harmful byproducts generated by plants under various abiotic stresses (such as salt, drought, high temperature, and intense light). Excessive accumulation leads to oxidative damage including lipid peroxidation, protein damage, and DNA strand breaks. Alternative splicing plays an important regulatory role in plant oxidative stress responses by modulating the expression of oxidative-stress-related genes. Under oxidative stress and hypoxia stress, the category distribution is as follows: Category II comprises SR45, and Category IV comprises AtU1A, whose direct role under oxidative stress has not been confirmed. All factors lack field validation. In the following, we evaluate each factor sequentially in the order of Category II, Category IV, and non-splicing factors. All factors lack field validation.

SR45 participates in high-light-induced oxidative stress responses through its two isoforms (SR45.1 and SR45.2); promoter analysis revealed that the SR45 promoter contains three light-responsive cis-elements as well as multiple MYC-responsive and ABA-responsive elements. Under high-light (HL) treatment, SR45 expression was significantly upregulated within 0.5–12 h. SR45 itself undergoes alternative splicing to generate two protein isoforms, SR45.1 and SR45.2, which exhibit distinctly different functions—only SR45.1 can reverse the anthocyanin hyperaccumulation phenotype of *sr45* mutants. Functional validation demonstrated that *sr45* mutants exhibit increased anthocyanin accumulation under high-light conditions and show enhanced tolerance to paraquat-induced oxidative stress [123]. In Arabidopsis, *AtU1A* influences ROS levels by directly binding to *ACO1* pre-mRNA and regulating its alternative splicing, and the *AtU1A* mutant is highly sensitive to salt stress, with increased ROS accumulation [27].

In terms of hypoxia, anaerobic germination of rice seeds occurs. Transcriptome analysis showed that hypoxia treatment induces a large number of differentially alternative spliced (DAS) events that do not overlap with differentially expressed genes (DEGs), with most AS events occurring independently of changes in gene expression levels, suggesting that AS has a unique function independent of transcriptional regulation during hypoxic germination [124] (Table 1).

In summary, the evidence chain for SR45 (Category II) under high-light stress is relatively complete, supported by experimental data on expression induction, generation of functionally divergent variants through its own alternative splicing, and the identification of some downstream target genes, and mutant oxidative stress tolerance phenotypes; however, the downstream target genes regulated by SR45 under high-light stress and the specific changes in their splicing events have not been systematically identified. The AtU1A-*ACO1* ROS regulatory mechanism has been validated under salt stress, but its direct role under oxidative stress requires further investigation. Transcriptomic analysis of rice hypoxic germination revealed that most DAS events do not overlap with DEGs, suggesting that AS may function independently of transcriptional regulation in hypoxic responses; however, this study remains at the level of AS profiling and lacks functional validation of specific splicing factors.

### 3.7. Heavy Metal Stress, Dark Stress, and Physiological and Developmental Interaction Networks Involving Splicing Factors

In the interaction networks involving heavy metal stress, dark stress, and the physiological and developmental processes mediated by splicing factors, the category distribution is as follows: Category I comprises the UAP56/COP1 interaction module; Category III comprises SR34b, RS40/RS41, OsSCL26 and AtSPF30; and Category IV comprises SRGs and PRMT5. Although the studies cover diverse stress types, the depth of mechanistic dissection varies considerably, with most factors remaining at the level of phenotypic associations or partial causal chains. In the following, we evaluate each factor in the order of Category I, Category III, Category IV, and non-splicing factor classes. All factors lack D7 field validation.

UAP56 and COP1 have been confirmed to physically interact through multiple experimental approaches, including yeast two-hybrid, BiFC, LCI, and Co-IP, to coordinately regulate alternative splicing of pre-mRNA. Knockdown of *UAP56* leads to enhanced photomorphogenesis, indicating that UAP56 is a negative regulator of photomorphogenesis. RNA-seq analysis revealed that UAP56 and COP1 co-regulate the expression of a large number of genes as well as alternative splicing events. Furthermore, RIP assays demonstrated that both UAP56 and COP1 are capable of binding U1, U2, U4, U5, and U6 snRNAs, as well as target mRNAs [54].

Semi-quantitative RT-PCR analysis showed that *AtSR34b* expression was upregulated in response to cadmium stress, and further RT-PCR analysis revealed that the *sr34b* mutant shows increased sensitivity to Cd, with splicing defects observed in the pre-mRNA of its target gene *IRT1* under Cd stress [125]. During de-etiolation of *Arabidopsis* seedlings, RS40 and RS41 function as positive regulators promoting cotyledon opening, and RT-PCR analysis revealed that light-induced alternative splicing changes were attenuated in the rs40 rs41 double mutant. The transcription of *RS41* is regulated by both light and ABA, and overexpression of *RS41* can overcome the inhibitory effect of endogenous ABA on cotyledon opening [126].

Rice OsSCL26 regulates phosphate homeostasis, affecting phosphorus uptake and distribution. The *Ossc126* mutant exhibited significantly higher phosphorus content than the wild-type under various phosphorus concentrations; qRT-PCR analysis revealed that upregulated transcript levels of multiple phosphorus transport-related genes, and qRT-PCR analysis showed significantly increased exon/intron retention ratios of *OsSPX-MFS2* and *OsNLA2* [7].

Transcriptome analysis showed that under dark conditions, the alternative splicing of splicing regulatory factors (SRGs) is preferentially regulated, while widespread changes in AS also occur in genes associated with photosynthesis and light signaling pathways, suggesting a potential link between the two [40]. Transcriptome analysis showed that *PRMT5* mutations lead to aberrant *PRR9* splicing, thereby affecting circadian period length [127]. The circadian clock not only regulates transcription but also modulates plant environmental adaptation through AS [128].

AtAFC2 kinase phosphorylates the SR protein RSZ21, regulating high-temperature-induced alternative splicing and thermomorphogenesis. The *afc2* mutant exhibits a more pronounced thermomorphogenesis phenotype under high-temperature conditions [76].

Meanwhile, seed dormancy and seedling light responses are among the most prominent developmental processes regulated by AS [129] []. The circadian clock regulates AS through the modulation of splicing factors: transcriptome sequencing has identified 31 splicing-related genes (SR proteins, hnRNPs, and spliceosome assembly factors) that exhibit rhythmic expression. *AtSPF30* mutants alter the amplitude and baseline levels of 32 rhythmic AS events without affecting the core circadian clock period [127]. Five intron retention (IR) variants of key photomorphogenic transcription factors (*BBX22*, *BBX24*, *PIF3*, *PIF4*, *HY5*) have been validated, with BBX22 positively regulating light-mediated hypocotyl elongation suppression [130]. IR variants regulate photomorphogenesis by encoding truncated proteins or forming heterodimers or homodimers with full-length proteins [130]. Rice OsbZIP1 generates two isoforms, OsbZIP1.1 and OsbZIP1.2; the former contains a complete COP1-binding domain and accumulates in the light while being degraded in the dark, whereas the latter lacks this domain and remains stable under both light and dark conditions, regulating photomorphogenesis [131].

In summary, splicing factors participate in heavy metal and dark stress responses at the transcriptional or alternative splicing levels and connect physiological and developmental networks through interactions with kinases (e.g., SnRK1-SRRM1L) and regulation of phosphate homeostasis (OsSCL26). AS regulation is particularly prominent in circadian clock and photomorphogenesis, with intron retention (IR) variants participating in photomorphogenic regulation by generating truncated proteins or forming heterodimers. UAP56, together with COP1, has established a relatively comprehensive regulatory chain (Category I), including direct RNA-binding evidence, experimentally validated splicing events, genetic causality, overexpression phenotypes, and partial transcriptomic associations. SR34b (Category III) includes experimentally validated splicing events, genetic causality, and partial transcriptomic associations, but lacks direct RNA-binding evidence and functional differences between isoforms. RS40/RS41 (Category III) includes experimentally validated splicing events, genetic causality, and partial overexpression phenotypes, but lacks direct RNA-binding evidence and functional differences between isoforms. OsSCL26 (Category III) includes experimentally validated splicing events and partial genetic causality. AtSPF30 (Category III) includes experimentally validated splicing events, genetic causality, and partial transcriptomic associations. SRGs and PRMT5 remain only at the level of transcriptomic associations (Category IV), lacking functional validation. AFC2, as a non-canonical splicing regulatory kinase, has been supported by phosphorylation evidence from in vitro kinase assays and by mutant phenotype validation, but it does not directly bind RNA, nor have the splicing events of its specific target genes been verified. With respect to non-splicing factors, the IR variants of five key photomorphogenesis-related transcription factors have been validated; among these, BBX22IR and BBX24IR regulate photomorphogenesis through heterodimerization, while rice OsbZIP1 produces two isoforms via alternative splicing, with clearly defined functional divergence. Notably, multiple stress response pathways ultimately converge on the regulation of splicing factor phosphorylation status (SnRK1 phosphorylation of SRRM1L, AFC2 phosphorylation of SR proteins, etc.), suggesting that phosphorylation modification may serve as a critical hub connecting upstream stress signals with downstream splicing regulation.

### 3.8. Cross-Talk Mechanisms in Stress Regulation

Synthesizing the regulatory roles of splicing factors and alternative splicing events across the different stress types described above, several common cross-stress features can be broadly summarized.

First, the regulatory modes of the splicing factors themselves exhibit three patterns: transcriptional upregulation (e.g., *SR45a* and *AtDGCR14L* under salt stress; *STA1* and *PICLN* under cold stress; *CYP18-2* under heat stress), generation of divergent variants through their own alternative splicing (e.g., *SR45a* under salt stress; *CiGI* under drought stress; *TaHSFA6e* and *ZmHsf23* under heat stress), and upstream kinase-mediated phosphorylation modification (e.g., GsSnRK1-GsSCL30a under alkaline stress; SnRK1-SRRM1L under salt stress; AFC2 under heat stress). All three patterns are observed across different stresses.

Second, different stress types may ultimately converge on similar biological pathways: the ABA signaling pathway serves as a common hub for splicing regulation under salt, drought, and alkaline stresses (SR45a, AtDGCR14L, RBM25, SmEb, SKIP, and AtU2AF65b all directly or indirectly target this pathway); ROS homeostasis is regulated by splicing factors under salt, oxidative, and high-light stresses; temperature stress predominantly involves splicing regulation of heat shock proteins and cold-adaptation genes.

However, the completeness of evidence varies among factors for each mechanism. Only a few cases—such as SR45a-*IDD14*, AtDGCR14L-*SWI3A*, SC35-*bZIP49*-AKT1, RBM25-*HAB1*, and CYP18-2—have established relatively complete causal chains. For most factors, functional characterization remains at the level of transcriptomic association or single-phenotype validation (e.g., PRP18, SR34b, RS40/RS41, AtSF1), with their direct targets and the biological functions of their splice variants yet to be identified. Furthermore, although *HvLHCA4.2*, *RVE2*, *P5CS1*, and *ZmHsf23* participate in stress responses through their own alternative splicing, they are not splicing factors themselves, but, rather, ‘target genes undergoing self-AS’ or ‘transcription factors undergoing self-AS’; the upstream splicing factors that regulate them remain unclear, indicating considerable scope for future investigation in this area.

## 4. Prospects for the Application of Splicing Factors in Plant Stress Tolerance Improvement

### 4.1. Current Challenges in Research

Although significant progress has been made in the study of plant splicing factors in abiotic stress responses over the past years, a considerable gap remains between translating this knowledge into effective crop stress tolerance improvement. Several key challenges in current research limit the application of splicing factors in practical breeding.

One prominent issue is the functional redundancy within splicing factor families. Splicing factors in plants typically exist as multigene families, with different members within the same gene family often exhibiting overlapping functions, which limits the effectiveness of improving traits through single-gene mutations. In tomato, RS2Z35 and RS2Z36 are two highly homologous plant-specific SR proteins, both of which suppress intron splicing of *HSFA2*. Single mutations in either RS2Z35 or RS2Z36 do not disrupt *HSFA2* splicing. In contrast, the RS2Z35/36 double homozygous mutants generated through crossing exhibit upregulated *HSFA2* splicing activity. This observation indicates that both proteins jointly negatively regulate HSFA2 intron splicing and exhibit functional redundancy with each other [117].

Another challenge is that overexpression of splicing factors often imposes a growth penalty on plants. Because splicing factors are broadly involved in RNA processing, their constitutive high-level expression can interfere with normal plant growth and development. In lily, although overexpression of the transcription factors *LlHSFA3A* and *LlHSFA3B* confers some improvement in thermotolerance, it leads to increased salt sensitivity, reduced thermotolerance under prolonged heat stress, and overall impairment of growth and development [132].

In Arabidopsis, constitutive overexpression of the splicing factor *SR45a* (particularly its two splice variants *SR45a-1a* and *SR45a-1b*) significantly reduces seed germination, cotyledon greening, and survival rates under salt stress, whereas *sr45a* knockout mutants conversely exhibit enhanced salt tolerance [96].

Furthermore, splicing factors exhibit pronounced tissue specificity and developmental stage dependence, rendering simple constitutive expression strategies ineffective. In Arabidopsis, *SR45a-1a* shows the highest expression levels in siliques and stems, whereas *SR45a-1b* is more highly expressed in roots and seeds [96]. In tea plants (*Camellia sinensis*), CYP18-2 shows a clear response to heat stress in mature leaves but exhibits little response in seedling leaves [116].

### 4.2. Future Application Prospects

**Figure 4:**
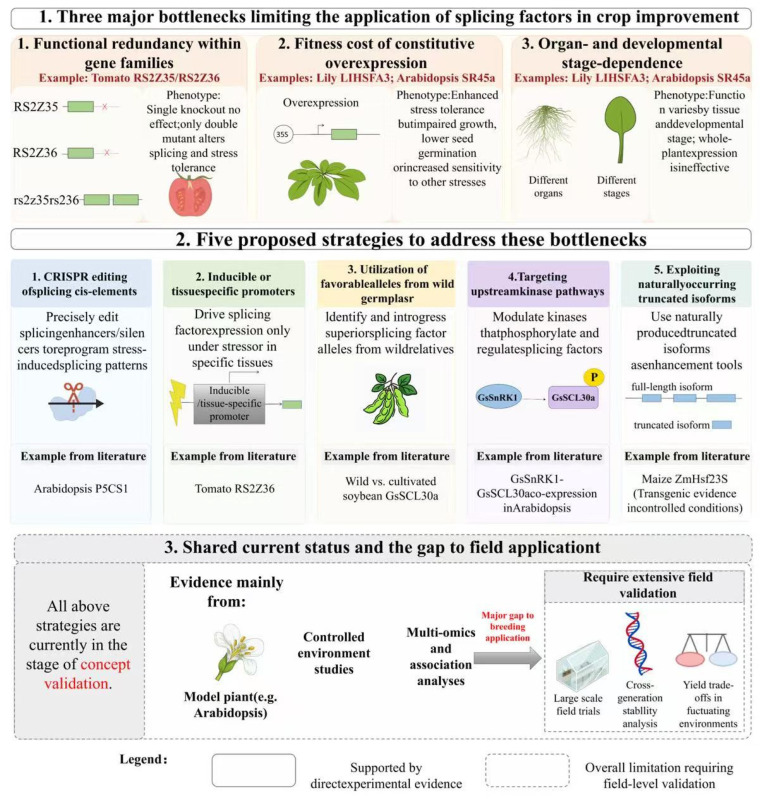
Bottlenecks, strategies, and validation status for the application of splicing factors in stress resistance breeding. This figure summarizes three experimentally validated bottlenecks (functional redundancy, growth penalty upon overexpression, and tissue/development dependence) and five categories of counter strategies (CRISPR editing, inducible promoters, utilization of wild alleles, kinase targeting, and enhancement of truncated isoforms), and indicates the current research endpoints for each strategy. All strategies are currently at the proof-of-concept stage, with evidence mainly derived from model plants or controlled environments; further field validation and yield assessment are required before they can be applied to breeding. Solid-line boxes indicate direct experimental evidence, whereas dashed-line boxes denote current research endpoints. Image was created with the tools of BioGDP (https://BioGDP.com (accessed on 26 July 2026)).

Despite the aforementioned challenges, the application of splicing factors in crop stress tolerance improvement remains a promising avenue. Based on existing research, the following directions may hold potential for practical application (Figure 4).

The first approach is precision editing of splice sites. Utilizing CRISPR/Cas9 technology for precise splice-site editing can alter stress-induced splicing patterns, thereby modulating the production ratio of functional isoforms. In Arabidopsis, natural variation in the number of TA repeats within intron 2 of the *P5CS1* gene leads to differences in exon 3 skipping frequencies, consequently affecting proline accumulation and climatic adaptation [114]. However, most current evidence derives from natural variation analyses in *Arabidopsis*; targeted splice-site editing in polyploid crops (such as wheat and cotton) remains technically challenging, and off-target effects on constitutive splicing have not been systematically evaluated.

The second approach involves the use of inducible or tissue-specific promoters. In tomato, *RS2Z36* is heat-inducibly expressed, whereas *RS2Z35* is constitutively expressed; their coordinated action enables plants to respond rapidly to heat stress while avoiding the growth penalty associated with sustained high-level expression [117]. Although the endogenous heat-inducible expression of *RS2Z36* provides a natural precedent, artificial inducible constructs have not yet been tested in stable transgenic crop lines, and leaky expression under non-stress conditions remains a concern requiring attention.

The third strategy is the mining and utilization of superior allelic variants from wild germplasm resources. In wild soybean, the expression level of the splicing factor *GsSCL30a* under alkaline stress is significantly higher than that of its homolog *GmSCL30a* in cultivated soybean [10]. In maize, the *ZmCCA1* gene produces three splice isoforms, among which *ZmCCA1.1* exhibits a relatively pronounced effect in drought tolerance, whereas the effect of *ZmCCA1.2* is negligible [133]. Although the *GsSCL30a* allele has been identified in wild soybean, its introgression into elite cultivars and its performance under multienvironment field trials have not yet been reported. Moreover, alleles that are beneficial in one genetic background often fail to produce equivalent effects in other backgrounds.

The fourth approach is targeting upstream regulatory kinases of splicing factors. In wild soybean, the activity of the splicing factor *GsSCL30a* is regulated by phosphorylation via the *GsSnRK1* kinase; co-overexpression of *GsSnRK1* and *GsSCL30a* exerts a synergistic effect in enhancing alkaline stress tolerance [10]. Kinase-targeted regulation has thus far been limited to co-overexpression of *GsSnRK1* and *GsSCL30a* in transgenic Arabidopsis. Whether this strategy is effective in soybean itself and whether it incurs growth penalties under normal conditions remain unknown.

The fifth strategy is the utilization of naturally occurring truncated isoforms as enhancing factors. In maize, although Hsf23L lacks intrinsic transcriptional activity on its own, it can interact with Hsf23S and enhance the latter’s ability to activate target genes; co-overexpression of Hsf23L and Hsf23S further improves thermotolerance in transgenic plants [120]. The synergistic effect of Hsf23L and Hsf23S has been observed in transgenic maize under controlled greenhouse conditions; validation of its practical value under fluctuating field conditions remains necessary.

In summary, the current application of splicing factors in stress tolerance improvement is primarily constrained by the following bottlenecks: functional redundancy among members of the same family limits the effectiveness of single-gene mutations, constitutive overexpression frequently impairs growth and development, and splicing factor functions exhibit pronounced tissue specificity and developmental stage dependence. In response to these challenges, multiple strategies have been proposed in existing studies, including the use of CRISPR/Cas9 for precision splice-site editing to alter stress-induced splicing patterns, the employment of stress-inducible or tissue-specific promoters to drive splicing factor expression and mitigate growth penalties, the mining of superior allelic variants from wild germplasm resources, the targeting of upstream regulatory kinases of splicing factors for coordinated regulation, and the utilization of naturally occurring truncated isoforms as enhancing factors to improve stress tolerance. These strategies address the obstacles in breeding applications of splicing factors from different perspectives. It must be emphasized that the aforementioned strategies remain at the proof-of-concept stage, having been validated primarily in model plants (such as Arabidopsis) or under controlled greenhouse conditions. Translating these findings into commercial breeding pipelines will still require extensive field trials, multigenerational stable inheritance analyses, and careful evaluation of yield trade-offs under fluctuating environmental conditions.

## 5. Summary and Perspectives

This review systematically summarizes the classification features, modes of action, and regulatory mechanisms of plant splicing factors as core spliceosomal components in abiotic stress responses, establishing that splicing factors participate in plant responses to multiple stresses through processes including splice-site recognition and the regulation of spliceosome assembly and disassembly. Current research remains largely concentrated on Arabidopsis and single-stress conditions; future efforts should be extended to major crops such as wheat, rice, and maize, and should strengthen investigations into regulatory mechanisms under complex stress combinations. Meanwhile, integrating gene editing and other technologies will help refine the molecular regulatory networks governing plant stress responses. Overall, plant splicing factors, as important ‘dynamic regulators’ that bridge environmental signals and gene expression, hold broad prospects for application in crop stress tolerance improvement. However, their practical application in breeding still faces challenges such as functional redundancy, growth penalties, and tissue-specificity issues. In the future, research should integrate strategies such as precision splicing editing, inducible expression systems, and the mining of superior allelic variants; yet, extensive field trials remain essential to facilitate the translation of fundamental research into field-based applications.

## Data Availability

No new data were created or analyzed in this study. Data sharing is not applicable to this article.
